# Rapid Degradation of SARS-CoV-2 Spike S Protein by A Specific Serine Protease

**DOI:** 10.3390/molecules27061882

**Published:** 2022-03-14

**Authors:** Jiankai Liu, Mujie Kan, Lianzhi Zhang, Yuan Yue, Shaohua Wang, Min Hong, Xinyu Hong

**Affiliations:** 1 Biochemistry Department, College of Basic Medical Sciences, Jilin University, Changchun 130021, China; liujk@jlu.edu.cn (J.L.); kanmj@jlu.edu.cn (M.K.); zlz@jlu.edu.cn (L.Z.); hongmin@jlu.edu.cn (M.H.); 2 Laboratory of Medical Biology Center, College of Basic Medical Sciences, Jilin University, Changchun 130021, China; yueyuan@jlu.edu.cn; 3 Institute of Virology and AIDS Research, The First Hospital of Jilin University, Changchun 130021, China; wangshaohua@jlu.edu.cn; 4 Neuroscience Research Laboratory, Neurosurgery Department, The First Hospital of Jilin University, Jilin University, Changchun 130021, China

**Keywords:** SARS-CoV-2, spike (S) protein, alkaline serine protease with an acidic pI (ASPNJ), degradation of S protein, antiviral agent

## Abstract

The S protein of SARS-CoV-2 is a crucial structural and functional component for virus entry. Due to the constant mutation of the virus, there are very limited ways to prevent and control COVID-19. This experiment used a macroscopic SDS-PAGE method and proved that the S protein of wild-type SARS-CoV-2 virus, especially the S1 subunit, is very sensitive to alkaline serine protease with acidic pI (ASPNJ), NJ represents *Neanthes japonica* (Izuka) from which ASP is purified). ASPNJ cleaves proteins when the carbonyl group of the peptide bond is contributed by arginine or lysine. ASPNJ can degrade the S protein very quickly and effectively in vitro with relative selectivity. It can be inferred that the S, S1 and RBD of SARS-CoV-2 variants can also be easily degraded by ASPNJ. This rapid and strong degradation of the S protein by ASPNJ may become a potential new treatment strategy.

## 1. Introduction

The Coronavirus Disease 2019 (COVID-19) pandemic, caused by Severe Acute Respiratory Syndrome Coronavirus 2 (SARS-CoV-2), has resulted in 276,436,619 confirmed cases with 5,374,744 deaths worldwide since first reported in December 2019 [1,2] (https://covid19.who.int/; accessed on 25 December 2021). Despite the significant progress made in developing vaccines against the disease, there are currently no specific treatments or approved drugs to treat infected people [3,4]. Vaccines and anti-SARS-CoV-2 monoclonal antibodies may be less effective against some of the new variants.

Heavily mutated SARS-CoV-2 strains continue to emerge in many regions. Based on the global tracking of SARS-CoV-2 mutations, the WHO classifies mutations into variants of concern (VOC), variants of interest (VOI), and variants under monitoring (VUM). VOC includes Alpha (B.1.1.7), Beta (B.1.351), Gamma (P.1), Delta (B.1.617.2), and Omicron B.1.1.529 (an emerging highly divergent variant with a large number of mutations related to immune escape potential and higher transmission) [5], while VOI includes Lambda (C.37) and Mu (B.1.621) as shown in Supplement Table S1. Certain mutations may change the characteristics and properties of the virus, leading to easier transmission of the virus, increased severity of diseases, reduced vaccine protection, heightened failure of antibody therapy, and escalated public health crisis [6,7,8,9,10].

The structures of many SARS-CoV-2 proteins have been revealed and deposited in the Protein Data Bank for the development of interventions against the disease [3]. Among several structural proteins, the spike (S) protein has received the most attention as a target for developing vaccines and drugs, primarily because the S protein plays an important functional element for the virus to enter the host cell [11]. Therefore, one promising therapeutic approach is to block the interaction between the S protein and ACE2 receptor using antibodies or small molecules [12,13]. On the other hand, the coagulation dysfunction observed in COVID-19 patients supports the biological rationale for anticoagulation therapy. As a result, there have been some reports on the application of heparin and synthetic heparin drugs to treat COVID-19, and they have shown better therapeutic effects or prognosis, compared to no treatment [14,15,16].

The alkaline serine protease with an acidic pI (ASPNJ) was previously purified from *Neanthes japonica* (Izuka) and characterized in our lab, formerly named NJF. ASPNJ has been demonstrated as a plasmin-like protease which can directly degrade fibrin and fibrinogen in vitro and in vivo [17,18]. The fibrinogenolysis pattern of this enzyme indicated that it preferentially hydrolyzed the Aα-chain in a very short time (1 min), and then the Bβ-chain within 10 min, while the γ-chain was completely degraded in 1 h. Further, it has been shown that ASPNJ can inhibit the proliferation of leukemia cells, induce leukemia cell apoptosis, and improve chemotherapy sensitivity [19]. The mechanism of the effects of ASPNJ on myeloid leukemia cells may involve the degradation of some membrane proteins or membrane associated proteins, as revealed by membrane proteomics [unpublished data]. As such, ASPNJ can degrade multiple substrates with different strengths and has potential for several applications. ASPNJ has strong amidolytic activity to the chromogenic substrates, S-2238, S-2444 and S-2251, indicating that the enzyme cleaves peptide bonds on the carbonyl side of arginine (Arg, R) and lysine (Lys, K) residues in proteins [17].

The amino acid sequence analysis of the wild-type SARS-CoV-2 S protein showed that the important domains and motifs in the S protein are rich in K and R residues. The receptor binding domain (RBD) of the S1 protein contains 23 K and R residues, and the percentage of these two basic amino acids to the total amino acids is 0.103 (23/223). The CendR (C-terminal rule) motif composed of RRAR is the C-terminal sequence of S1 after furin cleavage. These basic amino acid residues are the structural components of the S protein that maintain the normal structure and function of the virus. For example, the polar interaction between K417 in RBD and D30 in ACE2 receptor is involved in virus receptor recognition [20], whereas the CendR motif of S1 recognizes, combines, and activates neuropilin (NRP1 and NRP2) receptors of the respiratory epithelial cell surface [21]. Once the K or R residues in these regions were degraded, the virus would fail to infect host cells, or the infectivity of the virus would be greatly reduced.

Although there are few reports on antiviral studies using proteases to degrade viral proteins, it was reported that host cells may naturally resist viral infection by interacting with some viral proteins through the proteasomal degradation system [22,23]. For example, there is a direct interaction between host ubiquitin (Ub)-specific protease 15 (USP15) and Nef; USP15 degrades not only Nef but also Gag, the HIV-1 structural protein, thereby significantly inhibiting HIV-1 replication. Motivated by this area of research, we sought to determine whether ASPNJ might degrade the viral S protein and at the same time, play a role in anticoagulant therapy.

This experiment used a simple, direct, and reproducible macroscopic SDS-PAGE method and proved that S and S1 protein of wild-type SARS-CoV-2 are very sensitive to ASPNJ and are easily degraded by this enzyme in vitro at a very low enzyme concentration. We further compared the structural characteristics of S, S1, and RBD of SARS-CoV-2 wild-type and variant strains and analyzed their trypsin cleavage characteristics. It can be inferred that S, S1, and RBD of SARS-CoV-2 variants can also be easily degraded by ASPNJ. This rapid and strong degradation of the S protein by ASPNJ or new drugs with ASPNJ-like activity may become a potential therapeutic strategy for treating COVID-19.

## 2. Results

### 2.1. Comparison of the Molecular Weight of SARS-CoV-2 Spike Protein (S Full Length, S1 Subunit and RBD-mouseFc)

**Figure 1:**
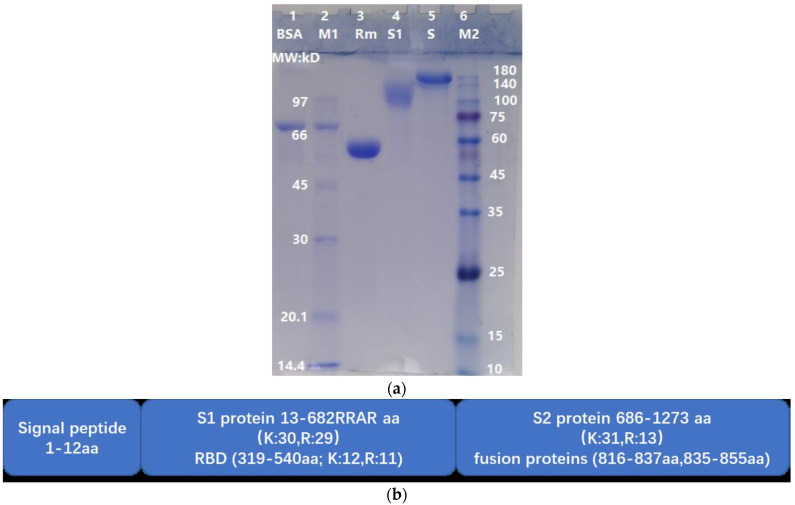
Molecular weight comparison of S, S1, and RBD-mouseFc by SDS-PAGE and schematic diagram of S protein. (**a**) Lane 1; Bovine serum albumin (BSA), MW 66 kD. Lane 2; MW marker 1 (M1). Lane 3; RBD-mouseFc (Rm), MW~55 kD. Lane 4; S1 protein (S1), approximately 110 kD. Lane 5; Spike protein(S), approximately 150 kD. Lane 6; MW marker 2 (M2). (**b**) S protein contains signal peptide (1–12 aa), S1 protein (13–685 aa) and S2 protein (686–1273 aa). RBD (319–540 aa) was a domain in S1 protein. The C-terminal sequence of S1 was 682RRAR, which was a specific structure motif, termed as C-end rule (CendR).

The theoretical molecular weights (MWs) of S, S1, and RBD of the wild-type strain are 141.2 kD, 75.3 kD and 25.1 kD, respectively. In this study, RBD-mouseFc was a fusion protein (RBD is recombinantly fused to the constant crystallizable fragment (Fc) of a mouse IgG molecule) with a theoretical MW of 51.5 kD. Due to glycosylation, the apparent MWs of S, S1, and RBD-mouseFc were greater than their theoretical MWs. As shown in Figure 1a, the apparent MWs of S, S1, and RBD-mouseFc were approximately 150 kD, 110 kD, and 55 kD, respectively. The undegraded (i.e., intact) S, S1, and RBD-mouseFc proteins were used as reference controls for observing the degradation effects of ASPNJ on each of the three proteins, respectively. The spike protein was 1273 aa in length (detail structure shown in Figure 1b).

### 2.2. Dose-Dependent Degradation of S, S1, and RBD-mouseFc Proteins by ASPNJ

**Figure 2:**
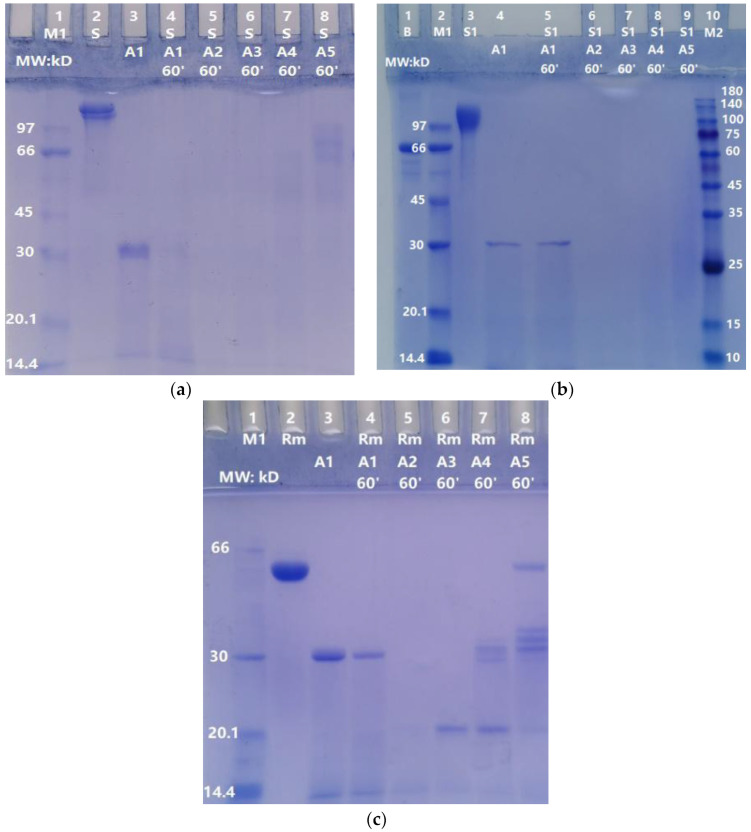
Degradation of S protein, S1 protein, and RBD-mouseFc by ASPNJ of different concentrations: (**a**) degradation of S; (**b**) degradation of S1; and (**c**) degradation of RBD-mouseFc (Rm). The concentration of S, S1, and Rm were all 10 μg/lane. A1–A5 were ASPNJ/lanes of different concentrations (A1–A5 were 50 (or 30), 1, 0.2, 0.04, 0.008 μg/lane, respectively). Each of the three proteins was degraded by ASPNJ, with a total volume of 25 μL, at 40 °C for 60 min, and the degradation effect was observed by running SDS-PAGE.

Different doses of ASPNJ (1 µg, 0.2 µg, 0.04 µg and 0.008 µg) were used to interact with the three proteins S, S1, and RBD-mouseFc (10 µg each) at 40 °C for 60 min in an Eppendorf tube, then the samples were subjected to SDS-PAGE, and the degradation of the three proteins by ASPNJ was observed. The results showed that higher degrees of degradation of the three proteins by ASPNJ were associated with higher concentrations of ASPNJ (Figure 2). High concentrations of ASPNJ (50 or 30 μg/lane) could completely hydrolyze 10 μg of S (lane 4 of Figure 2a), S1 (lane 5 of Figure 2b), and RBD-mouseFc (lane 4 of Figure 2c) proteins, respectively, within 60 min. As shown, the amount and MW of the remaining degradation residues increased with increasing dilution of ASPNJ. ASPNJ at a concentration as low as 0.008 µg can almost completely degrade 10 µg of S (lane 8 in Figure 2a) or S1 (lane 9 in Figure 2b) protein, leaving only a trace amount of incompletely hydrolyzed S or S1 protein. The ratio of S or S1 to ASPNJ exceeded 1000 times. However, the concentration of ASPNJ required for almost complete degradation of RBD-mouseFc was 1 µg (Figure 2c, Lane 5) instead of 0.008 µg. This may be because the RBD-mouseFc protein used here was a fusion protein, not an original RBD. Therefore, compared with the effect of ASPNJ on RBD-mouseFc, ASPNJ has a stronger observed hydrolysis effect on the S1 and S protein, especially on the S1 protein.

### 2.3. Time-Dependent Degradation of S, S1, and RBD-mouseFc Proteins by ASPNJ

**Figure 3:**
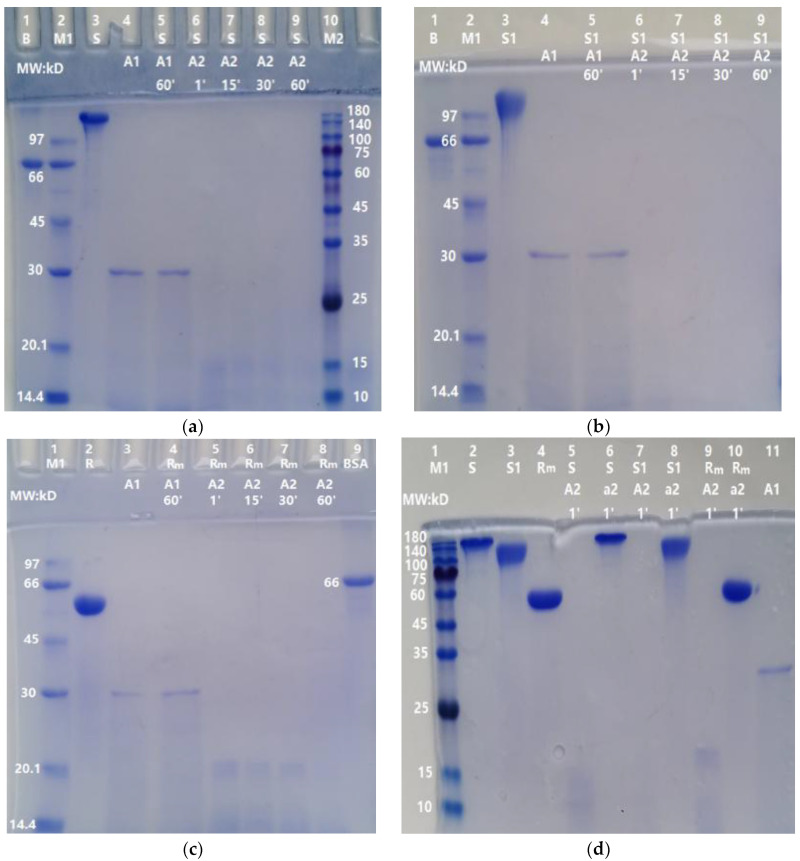
Degradation of S, S1, and Rm by ASPNJ for different incubation times: (**a**) degradation of S; (**b**) degradation of S1; (**c**) degradation of RBD-mouseFc (Rm); and (**d**) degradation of S, S1 and Rm by ASPNJ for 1 min. The concentrations of S, S1, and Rm were all 10 μg/lane. A1 and A2 were ASPNJ of different concentrations (A1–A2 was 30, 1 μg/lane). B: BSA. The three proteins were degraded with ASPNJ with a total volume of 25 μL, and degraded at 40 °C for 1, 15, and 30 to 60 min, respectively. The degradation effect was observed by running SDS-PAGE. The lowercase letter a in (**d**) represents the denatured ASPNJ, which was heated at 80 °C for 5 min. Both of A2 and a2 were 1 μg/lane.

As shown in Figure 3, the degradations of S, S1, and RBD-mouseFc (10 µg/lane) by ASPNJ (1 µg/ lane) occurred in a time-dependent manner, from 1 min, 15 min, and 30 min to 60 min. The degradation of the three proteins by 1 μg ASPNJ was almost completed within one minute (lane 6 in Figure 3a, lane 6 in Figure 3b, and lane 5 in Figure 3c, respectively), and the denatured ASPNJ completely lost its degradation action on S, S1, and RBD-mouseFc (lane 5, lane 7, and lane 9 in Figure 3d, respectively). The remaining degradation residues were barely visible with increasing time, but some smaller MW degradation residues could be observed with shorter incubation times. At the same time, we observed that S1 protein is more sensitive to ASPNJ, and the hydrolysis effect of ASPNJ on S1 protein is strong compared to that for the other two proteins.

### 2.4. Much Lower Degradation Effect of ASPNJ on Bovine Serum Albumin, BSA

**Figure 4:**
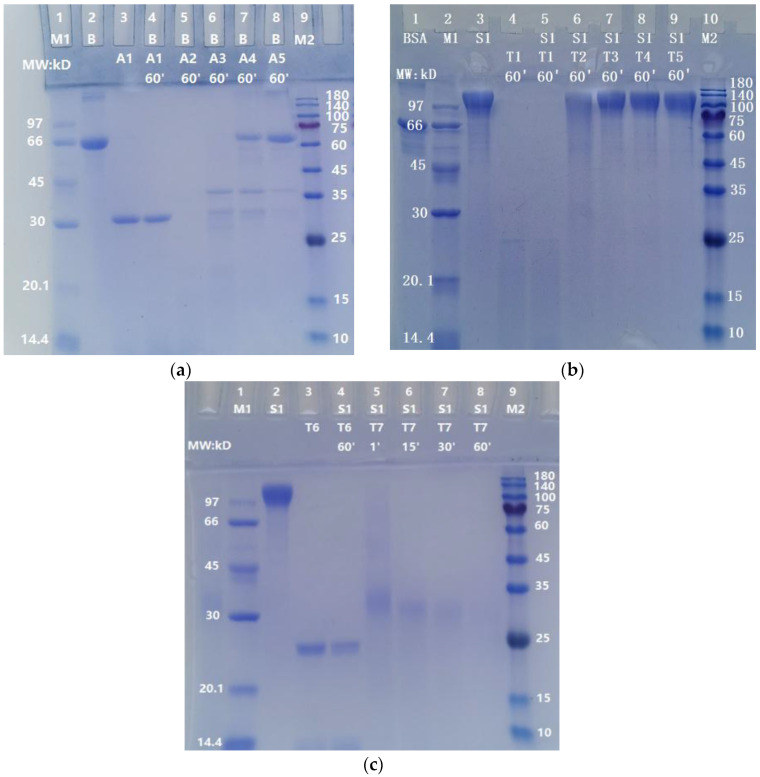
The action of ASPNJ on BSA and the action of trypsin on S1 protein. (**a**) BSA was degraded with ASPNJ with a total volume of 25 μL and degraded at 40 °C for 60 min. A: ASPNJ of different concentrations (A1–A5 were 50, 1, 0.2, 0.04 and 0.008 μg/lane, respectively). B: BSA (10 μg/lane). (**b**) Separate degradation reactions of different concentrations of trypsin to S1 protein, with a total volume of 25 μL, were carried out at 40 °C for 60 min. (**c**) Individual degradation reactions of S1 protein by trypsin occurred at different incubation times from 1, 15, 30 to 60 min. S1 protein was 10 μg/lane. T: Trypsin at different concentrations (50, 1, 0.2, 0.04, 0.008, 100 and 5 μg/lane for T1–T7, respectively). The degradation effects were observed by running SDS-PAGE.

In order to determine whether ASPNJ has a certain selectivity in the degradation of the S protein, BSA was used as an example and degraded in parallel by ASPNJ. As shown in Figure 4a, 1 μg ASPNJ could degrade 10 μg BSA, whereas 0.04 μg and 0.008 μg ASPNJ could not degrade all 10 μg intact BSA (lane 7 and 8 in Figure 4a). Comparing Figure 4a with Figure 2a,b, under the same conditions, 0.008 μg ASPNJ could completely degrade 10 μg S or 10 μg S1. These results revealed that the degradation ability of ASPNJ to S1 is stronger than that of BSA (approximately 125 times stronger).

### 2.5. Weak Degradation Effect of Trypsin on S1 Protein

To compare whether the proteolytic effect of ASPNJ on the S protein is stronger than that of trypsin, S1 protein was used as an example. The S1 subunit (10 µg) was degraded with different doses of trypsin (50 μg, 1 µg, 0.2 µg, 0.04 µg and 0.008 µg) for 60 min. The results showed that the S1 subunit (10 µg) could be completely degraded with trypsin at 50 μg (lane 5 in Figure 4b) and 100 μg (lane 4 in Figure 4c). However, the same degradation was very weak or almost invisible (lane 6 to lane 9 in Figure 4b) when the trypsin concentration was 1 µg or less than 1 µg. About 5 µg trypsin and 60 min were required for the almost complete degradation of 10 µg S1 (lane 8 in Figure 4c), whereas only 1 µg ASPNJ and one minute was required for the almost complete degradation of 10 µg S1 (lane 6 in Figure 3b). These results indicate that ASPNJ is far superior than trypsin in degrading the S1 protein.

### 2.6. The Structural Characteristics of SARS-CoV-2 Spike Protein and the Inference of ASPNJ Activity

The structural characteristics and properties of the wild-type and variants of SARS-CoV-2 were calculated and compared, as shown in the Supplement Table S1. The isoelectric point (pI), the trypsin cleavage site (CS), and non-trypsin cleavage site (NCS) of variants varied, but the number and location of most K and R were largely conserved in all virus strains, and as predicted, most of the mutations with R and K can be cleaved by trypsin. Since trypsin and ASPNJ have similar catalytic specificities, ASPNJ may also act on the spike proteins, S1 and, RBD of other SARS-CoV-2 variants.

## 3. Discussions

SARS-CoV-2 infection is triggered by ACE2 receptor binding and subsequent membrane fusion, followed by the release of the viral genome into the cell cytoplasm for replication. The process of virus binding and fusion are mediated by the S protein. During the binding process between the RBD of the S1 protein and the peptidase domain (PD) of the ACE2 receptor, the sequential cleavage of the site between S1 (685R) and (686S) S2 by the host proteases is critical for virus infection [24].

In this study, we have reported that in vitro ASPNJ can degrade the S, S1, and RBD-mouseFc proteins. And this degradation effect to these proteins of the SARS-CoV-2 wild-type strain is dependent on the application dose and action time of ASPNJ. ASPNJ can degrade spike protein(s) more simply and efficiently than trypsin. (lane 6 in Figure 3b vs. lane 8 in Figure 4b). Once ASPNJ was added to the S and S1 substrate solution, the degradation reaction took place immediately. Compared with S and S1, the degradation effect of ASPNJ on BSA was much weaker (Figure 4 vs. Figure 2a,b). A comprehensive comparison of the degradation effects of ASPNJ and trypsin on the S protein and BSA revealed that ASPNJ has a strong and rapid degradation capacity on S1 protein with relative specificity.

The Spike protein of wild-type SARS-CoV-2 was 1273 aa in length [2,25], containing 103 K and R residues. The prediction using the ExPASy peptide cutter and our experimental results showed that 101 R and K sites could be hydrolyzed by trypsin and ASPNJ, except for 462KP (P, proline, pro) and 811KP (Supplement Table S1). The 11 K sites (including 417K) and 11 R sites in RBD of S1, as well as the 682R, 683R, and 685R in the CendR motif of S1 and the 825K, 835K, 847R, and 854K in the fusion peptide of S2, can all be hydrolyzed by ASPNJ. If these sites in these domains and motifs are cleaved, the virus will lose the ability to recognize, bind, and activate the ACE2 and neuropilin (NRP1 and NRP2) receptors on the surface of respiratory epithelial cells, resulting in no membrane fusion and no viral genome entry [20,21]. The virus will not be able to infect host cells.

ExPASy PeptideCutter software analysis and our experimental results showed that RBD, S1, and S are sensitive to ASPNJ. The pI of ASPNJ is 4.4, whereas the pI of RBD, S1, and S are 8.91, 8.24, and 6.24, respectively. These properties may enhance the binding affinity between the S protein and ASPNJ through electrostatic interaction, similar to the binding between RBD and ACE2 receptors [26,27], which is conducive to the rapid hydrolysis and degradation of RBD and S1. Studies have shown that in the spike protein trimer particles, 1 erect and 2 erect RBD accounted for 16% and 4% of all RBD, respectively. These two conformations are open structures [28]. We speculate that bonds between the erect RBD before the SARS-CoV-2 trimer and ACE2 receptor may be more susceptible to degradation by ASPNJ.

At present, COVID-19 caused by the SARS-CoV-2 Delta and Omicron variants is surging globally. The Delta variant became the internationally dominant variant at the end of 2020, but it has gradually receded in some countries, replaced by the rapidly spreading Omicron variant. Due to the proliferation of the Omicron variant and its high resistance to neutralization by existing vaccines, it has become the variant of concern (VOC) since late 2021 [29]. It is speculated that the Omicron variant has a higher affinity for the ACE2 receptor than the Delta variant because of its mutation in the receptor-binding domain (RBD), leading to a higher transmission potential [30]. We calculated and analyzed the characteristics and properties of the S protein of 5 VOCs and 2 VOIs (Supplement Table S1). The mutations in the aa sequence of various virus strains did not significantly change the structural features and characteristics related to trypsin and ASPNJ. Non-aa or few aa mutations in the variant S protein significantly affect the number of non-cleavage sites (NCS) and the K and R positions for trypsin and ASPNJ cleavage. Most mutations, such as E484 K in Beta, Gamma and Mu variants, T19R, L452R and P681R in the Delta variant, and Q493R, T547K, N679K, N764K, N856K, and N969K in the Omicron variant can be cleaved by trypsin or ASPNJ. Only a few mutations to K or R in variants could not be cleaved by trypsin or ASPNJ (e.g., the T478K mutation in the Delta variant could not be cut due to 479P steric hindrance; and the Q498R mutation in the Omicron variant could not be cut due to 499P steric hindrance). Therefore, the aa mutation of the variants did not significantly increase the number of NCS in the S protein, especially in the RBD and CendR motifs. At the same time, the aa mutation of the S protein of different variants increased the pI of the S, S1, and RBD regions. The increased pI can enhance the polar interaction between RBD and ACE2 receptors, but it also enhances the interaction between the S protein and ASPNJ, thereby promoting the degradation of S, S1, and RBD by ASPNJ. Therefore, just as ASPNJ acts on the SARS-CoV-2 wild-type strain, it may similarly degrade the S or S1 protein of variants.

ASPNJ has the advantages of low toxicity to normal host cells as well as no hemolysis or agglutination of human red blood cells in vivo and in vitro [18,19]. In intravenous injection experiments, no toxicity or damage was observed to the liver, kidney, and cardiovascular system of mice, rats, rabbits, and beagle dogs, and the LD50 of mice was 20 mg/kg body weight (unpublished data). In addition, ASPNJ is relatively stable. The enzyme activity is stable in the range of 40~80 °C and pH 6~11. Its maximum enzymatic activity was observed at 60 °C and pH 9.0. ASPNJ was purified and stored as a lyophilized powder at −20 °C. Its fibrinolytic activity can even be maintained for more than 10 years. We hypothesize that ASPNJ in vivo experiments may degrade the virus S protein and at the same time, it may be beneficial to the improvement of patients with coagulation dysfunction.

## 4. Materials and Methods

### 4.1. Viral Proteins of SARS-CoV-2

Three viral proteins were used in our experiments: recombinant SARS-CoV-2 spike protein **S** full length (CAS: VISC2-S002); recombinant SARS-CoV-2 spike protein S1 subunit (CAS: E007); recombinant SARS-CoV-2 spike protein RBD-mouseFc (CAS: E006). All viral proteins were expressed in host cell HEK293 with purity > 90% as determined by SDS-PAGE. The proteins were prepared by Jiangsu East-mab Biomedical Technology Co. Ltd. and obtained from the Shanghai HuicH Biotech Co. Ltd. (Shanghai, China).

### 4.2. Alkaline Serine Protease with an Acidic pI (ASPNJ) and Concentration Determination

ASPNJ was extracted and characterized as having a molecular weight (MS) of approximately 28 kD and a pI of 4.4. The maximum activity of the enzyme was observed at 60 °C and pH 9.0. The estimated fibrinolytic activity was 50,000 IU/mg, using the fibrin plate method. ASPNJ can degrade the chromogenic substrates S-2238 (H-D-Phe-Pip-Arg-pNA), S-2444 (pyro-Glu-Gly-Arg-pNA) and S-2251 (H-D-Val-Leu-Lys-pNA), indicating the specificity of the enzyme for arginine and lysine residues on the carbonyl side [14,17]. After comparing several different concentration determination methods, we found that the A280/A260 method was closest to the Kjeldahl method. Therefore, the concentration of ASPNJ in solution was estimated using the A280/A260 method and calculated as concentration (mg/mL) = (1.45 × A280) − 0.74 × A260) [31].

### 4.3. Other Reagents

Bovine serum albumin (BSA) was purchased from Sigma Chemical Co. (Burlington, MA, USA) (Prod. No. A-7517). Trypsin was purchased from Amresco ([0458], CAS# 9002-07-7). Low molecular weight calibration kits for SDS electrophoresis (molecular weight marker 1, M1), code: 17-0446-01, was purchased from Amersham Pharmacia Biotech (Hong Kong, China) (MW: 97 kD, 66 kD, 45 kD, 30 kD, 20.1 kD, 14.4 kD, respectively). The GoldBand 3-color Regular Range Protein Marker (molecular weight marker 2, M2) was provided by WuHan YEASEN Biotechnology Co., Ltd. (Wuhan, China) (MW: 180 kD, 140 kD, 100 kD, 75 kD, 60 kD, 45 kD, 25 kD, 15 kD, 10 kD, respectively). SDS-PAGE Gel Kit, Cat No: PH0331, was provided by Phygene Life Sciences Co., Ltd. (Fuzhou, China).

### 4.4. SARS-CoV-2 Spike Protein (S Full Length, S1 Subunit and RBD-mouseFc) Degradation and SDS-PAGE Observation

Each lyophilized powder was initially prepared from a 1 mL volume of 10 mM phosphate buffered saline (PBS) pH 7.4 at a protein concentration of 1 mg/mL. PBS (1×) consists of 137 mM NaCl, 10 mM phosphate (8 mM Na_2_HPO_4_ and 2 mM KH_2_PO_4_), and 2.7 mM KCl. These lyophilized protein substrates (S full length, S1 subunit, RBD-mouseFc and BSA) were separately dissolved in 0.5 mL deionized water according to the manufacturer’s instructions to make each substrate stock solution at a concentration of 2 mg/mL, PBS is (2×). The lyophilized ASPNJ and trypsin were separately dissolved in physiological saline. The degradation reaction of the substrate by ASPNJ or trypsin was carried out at 40 °C with a total volume of 25 μL. Each reaction contained 5 μL substrate (10 μg) and 20 μL enzyme solution with different concentrations, as shown in the figure legends. Samples were denatured at 80 °C for 5 min before running the sodium dodecyl sulfate-polyacrylamide gel electrophoresis (SDS-PAGE).

SDS-PAGE was performed according to methods previously reported [32], using 5% stacking and 12% resolving polyacrylamide gel. The gels were stained with Coomassie Brilliant Blue R250 (Sigma Aldrich) and examined.

### 4.5. Comparison of the Structural Characteristics and Properties of SARS-CoV-2 Wild-Type and Variant Strains

The aa sequence data of the spike protein, S1, and RBD were quoted from three online databases (https://www.uniprot.org/ (accessed on 18 July 2020), https://cov-lineages.org (accessed on 29 October 2021) and https://outbreak.info (accessed on 21 December 2021)). The isoelectric point (pI) and the number of cleavage sites (CS) and non-cleavage sites (NCS) of the three proteins by trypsin were calculated using https://web.expasy.org/compute_pi/ (accessed on 21 December 2021) and https://web.expasy.org/peptide_cutter/ (accessed on 21 December 2021).

Trypsin is known to preferentially cleave peptide bonds on the carbonyl side of arginine (Arg, R) and lysine (Lys, K) residues in protein. This catalytic specificity is also shared by ASPNJ. The possible degradation effect of ASPNJ on S, S1, and RBD from different SARS-CoV-2 variant strains were deduced by comparing the structural characteristics and properties of SARS-CoV-2 wild-type and variant strains.

## 5. Conclusions

Our experiments proved that ASPNJ can degrade the spike protein of the SARS-CoV-2 wild-type strain very quickly and effectively in vitro, and the degradation effect of ASPNJ is relatively specific for S and S1. At the same time, theoretical analysis shows that ASPNJ may also have a similar degradation effect on SARS-CoV-2 variants. We hope this research can potentially lead to new methods or directions for developing treatment for diseases caused by SARS-CoV-2.

## Supplementary Materials

The following supporting information can be downloaded at: https://www.mdpi.com/article/10.3390/molecules27061882/s1, Table S1: Comparison of the structural characteristics of SARS-CoV-2 wild-type and variants and the prediction of the effect of ASPNJ on S protein.

## Sample Availability Statement

ASPNJ is available in small quantities.

## References

[1] Zhu N., Zhang D., Wang W., Li X., Yang B., Song J., Zhao X., Huang B., Shi W., Lu R. (2020). A Novel Coronavirus from Patients with Pneumonia in China, 2019. N. Engl. J. Med..

[2] Wu F., Zhao S., Yu B., Chen Y.-M., Wang W., Song Z.-G., Hu Y., Tao Z.-W., Tian J.-H., Pei Y.-Y. (2020). A new coronavirus associated with human respiratory disease in China. Nature.

[3] Imran M. (2020). The SARS-CoV-2 Spike Glycoprotein as a Drug and Vaccine Target: Structural Insights into Its Complexes with ACE2 and Antibodies. Cells.

[4] Samrat S.K., Tharappel A.M., Li Z., Li H. (2020). Prospect of SARS-CoV-2 spike protein: Potential role in vaccine and therapeutic development. Virus Res..

[5] Minol (2021). Enhancing Readiness for Omicron (B.1.1.529): Technical Brief and Priority Actions for Member States. Zenodo.

[6] Wang R., Zhang Q., Ge J., Ren W., Zhang R., Lan J., Ju B., Su B., Yu F., Chen P. (2021). Analysis of SARS-CoV-2 variant mutations reveals neutralization escape mechanisms and the ability to use ACE2 receptors from additional species. Immunity.

[7] Ong S.W.X., Chiew C.J., Ang L.W., Mak T.-M., Cui L., Toh M.P.H.S., Lim Y.D., Lee P.H., Lee T.H., Chia P.Y. (2021). Clinical and Virological Features of Severe Acute Respiratory Syndrome Coronavirus 2 (SARS-CoV-2) Variants of Concern: A Retrospective Cohort Study Comparing B.1.1.7 (Alpha), B.1.351 (Beta), and B.1.617.2 (Delta). Clin. Infect. Dis..

[8] Korber B., Fischer W.M., Gnanakaran S., Yoon H., Theiler J., Abfalterer W., Hengartner N., Giorgi E.E., Bhattacharya T., Foley B. (2020). Tracking Changes in SARS-CoV-2 Spike: Evidence that D614G Increases Infectivity of the COVID-19 Virus. Cell.

[9] Mallavarpu Ambrose J., Priya Veeraraghavan V., Kullappan M., Chellapandiyan P., Krishna Mohan S., Manivel V.A. (2021). Comparison of Immunological Profiles of SARS-CoV-2 Variants in the COVID-19 Pandemic Trends: An Immunoinformatics Approach. Antibiotics.

[10] Hoffmann M., Arora P., Groß R., Seidel A., Hörnich B.F., Hahn A.S., Krüger N., Graichen L., Hofmann-Winkler H., Kempf A. (2021). SARS-CoV-2 variants B.1.351 and P.1 escape from neutralizing antibodies. Cell.

[11] Tse L.V., Meganck R.M., Graham R.L., Baric R.S. (2020). The Current and Future State of Vaccines, Antivirals and Gene Therapies Against Emerging Coronaviruses. Front. Microbiol..

[12] Barnes C.O., Jette C.A., Abernathy M.E., Dam K.-M.A., Esswein S.R., Gristick H.B., Malyutin A.G., Sharaf N.G., Huey-Tubman K.E., Lee Y.E. (2020). SARS-CoV-2 neutralizing antibody structures inform therapeutic strategies. Nature.

[13] Elshabrawy H.A. (2020). SARS-CoV-2: An Update on Potential Antivirals in Light of SARS-CoV Antiviral Drug Discoveries. Vaccines.

[14] Giannis D., Ziogas I.A., Gianni P. (2020). Coagulation disorders in coronavirus infected patients: COVID-19, SARS-CoV-1, MERS-CoV and lessons from the past. J. Clin. Virol..

[15] Lindahl U., Li J. (2020). Heparin—An old drug with multiple potential targets in Covid-19 therapy. J. Thromb. Haemost..

[16] Yang Y., Du Y., Kaltashov I.A. (2020). The Utility of Native MS for Understanding the Mechanism of Action of Repurposed Therapeutics in COVID-19: Heparin as a Disruptor of the SARS-CoV-2 Interaction with Its Host Cell Receptor. Anal. Chem..

[17] Deng Z., Wang S., Li Q., Ji X., Zhang L., Hong M. (2010). Purification and characterization of a novel fibrinolytic enzyme from the polychaete, Neanthes japonica (Iznka). Bioresour. Technol..

[18] Wang S.-H., Li Q., Deng Z.-H., Ji X., Jiang X., Ge X., Bo Q.-Q., Cui J.-Y., Zhang L.-Z., Liu J.-K. (2011). Neanthes japonica (Iznka) fibrinolytic enzyme reduced cerebral infarction, cerebral edema and increased antioxidation in rat models of focal cerebral ischemia. Neurosci. Lett..

[19] Ge X., Bo Q., Hong X., Cui J., Jiang X., Hong M., Liu J. (2013). A novel acidic serine protease, ASPNJ inhibits proliferation, induces apoptosis and enhances chemo-susceptibility of acute promyelocytic leukemia cell. Leuk. Res..

[20] Yan R., Zhang Y., Li Y., Xia L., Guo Y., Zhou Q. (2020). Structural basis for the recognition of SARS-CoV-2 by full-length human ACE2. Science.

[21] Daly J.L., Simonetti B., Klein K., Chen K.-E., Kavanagh Williamson M., Antón-Plágaro C., Shoemark D.K., Simón-Gracia L., Bauer M., Hollandi R. (2020). Neuropilin-1 is a host factor for SARS-CoV-2 infection. Science.

[22] Pyeon D., Timani K.A., Gulraiz F., He J.J., Park I.-W. (2016). Function of ubiquitin (Ub) specific protease 15 (USP15) in HIV-1 replication and viral protein degradation. Virus Res..

[23] Pyeon D., Rojas V.K., Price L., Kim S., Singh M., Park I.-W. (2019). HIV-1 Impairment via UBE3A and HIV-1 Nef Interactions Utilizing the Ubiquitin Proteasome System. Viruses.

[24] Zhang Q., Xiang R., Huo S., Zhou Y., Jiang S., Wang Q., Yu F. (2021). Molecular mechanism of interaction between SARS-CoV-2 and host cells and interventional therapy. Signal Transduct. Target. Ther..

[25] Zhang J., Xiao T., Cai Y., Chen B. (2021). Structure of SARS-CoV-2 spike protein. Curr. Opin. Virol..

[26] Zhou T., Tsybovsky Y., Gorman J., Rapp M., Cerutti G., Chuang G.-Y., Katsamba P.S., Sampson J.M., Schön A., Bimela J. (2020). Cryo-EM Structures of SARS-CoV-2 Spike without and with ACE2 Reveal a pH-Dependent Switch to Mediate Endosomal Positioning of Receptor-Binding Domains. Cell Host Microbe.

[27] Qiao B., de la Cruz M.O. (2020). Enhanced Binding of SARS-CoV-2 Spike Protein to Receptor by Distal Polybasic Cleavage Sites. ACS Nano.

[28] Benton D.J., Wrobel A.G., Xu P., Roustan C., Martin S.R., Rosenthal P.B., Skehel J.J., Gamblin S.J. (2020). Receptor binding and priming of the spike protein of SARS-CoV-2 for membrane fusion. Nature.

[29] Ghosh N., Nandi S., Saha I. (2022). A review on evolution of emerging SARS-CoV-2 variants based on spike glycoprotein. Int. Immunopharmacol..

[30] Kumar S., Thambiraja T.S., Karuppanan K., Subramaniam G. (2021). Omicron and Delta variant of SARS-CoV-2: A comparative computational study of spike protein. J. Med. Virol..

[31] Stoscheck C.M. (1990). Quantitation of protein. Methods Enzymol..

[32] Brunelle J.L., Green R. (2014). One-dimensional SDS-Polyacrylamide Gel Electrophoresis (1D SDS-PAGE). Methods Enzymol..

