# Supplementary material for: Rapid Degradation of SARS-CoV-2 Spike S Protein by A Specific Serine Protease
Source: Molecules. 2022 Mar 14;27(6):1882. doi: 10.3390/molecules27061882 (PMC8954242; doi:10.3390/molecules27061882)
Supplement: Supplementary file 1 [file molecules-27-01882-s001.zip › manuscript-supplementary.pdf]

Supplement Table S1. Comparison of the structural characteristics of SARS-CoV-2 wild and variants and the prediction of the effect of ASPNJ on S protein

			1	2	3	4	5	6	7
	WHO<br>PANGO	Wild<br>wild	Alpha<br>B.1.1.7	Beta<br>B.1.351	Gamma<br>P.1	Delta<br>B.1.617.2	Omicron<br>BA.1	Lambda<br>C.37	Mu<br>B.1.621
S	aa mutations of S protein		del69/70(HV),<br>del144(Y),<br>N501Y,<br>A570D,<br>D614G,<br>P681H,<br>T716I,<br>S982A,<br>D1118H	D80A,<br>D215G,<br>K417N,<br>E484K,<br>N501Y,<br>D614G,<br>A701V	L18F,<br>T20N,<br>P26S,<br>D138Y,<br>R190S,<br>K417T,<br>E484K,<br>N501Y,<br>D614G,<br>H655Y,<br>T1027I,<br>V1176F	T19R,<br>E156G,<br>del157/158<br>(FR),<br>L452R,<br>T478K,<br>D614G,<br>P681R,<br>D950N	A67V,del69/70(HV),T95I,G142D,<br>del143/145(VYY),N211I,del212/212(L),<br>ins214(EPE),G339D,S371L,S373P,S375F,<br>Q493R,G496S,Q498R,N501Y,Y505H,T547K<br>,D614G,H655Y,N679K,P681H,N764K,<br>D796Y,N856K,Q954H,<br>N969K,L981F	G75V,<br>T76I,<br>R246N,<br>del247/253，<br>(SYLTPGD)<br>L452Q,<br>F490S,<br>D614G,<br>T859N,	T95I,<br>Y145N,<br>R346K,<br>E484K,<br>N501Y,<br>D614G,<br>P681H,<br>D950N
	aa length/pI	1261/6.23	1258/6.35	1261/6.63	1261/6.38	1259/6.93	1258/6.79	1254/6.32	1261/6.66
	No. of K/R	61/42	61/42	61/42	61/41	62/44	66/44	61/41	63/41
	No. of CS/<br>No. of NCS	101/2	101/2	101/2	100/2	102/4	107/3	100/2	102/2
	PNCS	462(*450)**K**P,<br>811(*799)**K**P	462(*447)**K**P,<br>811(*796)**K**P	462(*450)**K**P,<br>811(*799)**K**P	462(*450)**K**P,<br>811(*799)**K**P	462(*448)**K**P,<br>478(*464)**K**P,<br>682(*668)**R**R<br>811(*797)**K**P	462(*447)**K**P,<br>498(*483)**R**P,<br>811(*796)**K**P	462(*443)**K**P,<br>811(*792)**K**P	462(*450)**K**P,<br>811(*799)**K**P
S1	aa length/pI	673/8.24	670 / 8.24	673/8.59	673/8.43	671/8.65	670/8.35	666/8.35	673/8.51
	No. of K/R	30/29	30/29	30/29	30/28	31/31	32/31	30/28	32/28
	No. of CS/<br>No. of NCS	58/1	58/1	58/1	57/1	59/2	61/2	57/1	59/1
	C terminal	669PRRAR	666HRRAR	669PRRAR	669PRRAR	667R**R**RAR	666HRRAR	662PRRAR	669HRRAR
RBD	aa length/pI	223/8.91	223/8.90	223/9.00	223/9.00	223/9.09	223/9.01	223/8.91	223/9.07
	No. of K/R	12/11	12/11	12/11	12/11	13/12	12/13	12/11	14/10
	No. of CS/<br>No. of NCS	22/1	22/1	22/1	22/1	23/2	23/2	22/1	23/1
S2	aa length/pI	588/5.25	588/5.39	588/5.25	588/5.25	588/5.32	588/5.58	588/5.25	588/5.32
	No. of K/R	31/13	31/13	31/13	31/13	31/13	34/13	31/13	31/13
	No. of CS/<br>No. of NCS	43/1	43/1	43/1	43/1	43/1	46/1	43/1	43/1

Columns 1-5 are Variants of Concern (VOC), and columns 6,7 are Variants of Interest (VOI) according to the WHO (World Health Organization) and PANGO (Phylogenetic assignment of the named global outbreak). S: Spike glycoprotein, S1: S1 protein, RBD: receptor binding domain. Amino acids (aa) in bold letters such as 462**K**P and 682**R**R indicate that they cannot be hydrolyzed by trypsin. No. of CS: theoretical No. of cleavage sites (CS) by trypsin; No. of NCS: theoretical No. of non-cleavage sites (NCS) by trypsin. PNCS: Trypsin's

theoretical position of NCS in S, S1 and RBD. *: The position of NCS in the mature sequence of S, S1 and RBD without signal peptide. No. of K/R: numbers of Lysine / number of Arginine. The c-terminal sequence RRAR of S1 protein, called CendR, can be hydrolyzed by trypsin, causing the c-terminal of S1 protein to fail to bind and activate neuropilin receptors (NRP1 and NRP2). The aa sequence data of S, S1 and RBD were quoted from https://www.uniprot.org/, https://cov-lineages.org and https://outbreak.inf. The pI, No. of CS and NCS by trypsin were calculated using https://web.expasy.org/compute_pi/ and https://web.expasy.org/peptide_cutter/. Trypsin preferentially cleaves the carbonyl side of arginine or lysine. Since this catalytic specificity is also shared by ASPNJ, this table predicts CS etc. using trypsin.
